# The effects for PM2.5 exposure on non-small-cell lung cancer induced motility and proliferation

**DOI:** 10.1186/s40064-016-3734-8

**Published:** 2016-12-01

**Authors:** Biao Yang, Dongmei Chen, Hui Zhao, Chunling Xiao

**Affiliations:** 1 Basic Discipline of Chinese and Western Integrative, Liaoning University of Traditional Chinese Medicine, Shenyang, 110032 Liaoning People’s Republic of China; 2 Key Lab of Environmental Pollution and Microecology, Shenyang Medical College, No. 146, Huanghe North Street, Shenyang, 110034 Liaoning Province People’s Republic of China

**Keywords:** Cell proliferation, Migration, Non-small cell lung cancer, PM2.5

## Abstract

**Background:**

Increasing urbanization and associated air pollution, including elevated levels of particulate matter (PM), are strongly correlated with the development of various respiratory diseases. In particular, PM2.5 has been implicated in promoting lung cancer initiation, growth and progression. Cell migration and proliferation are crucial for the progression of cancer. However, the molecular signatures and biological networks representing the distinct and shared features of non-small cell lung cancer (NSCLC) after PM2.5 exposure are unknown.

**Results:**

Functional assays demonstrated higher proliferation, migration and invasion of cancer cells stimulated with PM2.5. To investigate the complicated mechanisms, we performed global transcriptome profiling of the A549 cell line. Particularly, transcriptome sequencing revealed invasive characteristics reminiscent of cancer cells. By comparing the transcriptomes, we identified distinct molecular signatures and cellular processes defining the invasive and proliferative properties of PM2.5-exposed cells, respectively. Interestingly, under the PM2.5-stimulated condition, the A549 and H1299 cells strengthened obviously properties in motility and proliferation. Based on the network model reconstructing the shared protein–protein interactions, we selected the two most up-regulated genes, interleukin-1β (IL1β) and matrix metalloprotease 1 (MMP1), as key regulators responsible for the effects of PM2.5 exposure. Notably, IL1β and MMP1 expression was elevated in independent assays, which was further enhanced by PM2.5.

**Conclusion:**

Taken together, our systems approach to investigating PM2.5 exposure provides a basis to identify key regulators responsible for the pathological features of NSCLC.

## Background

Rapid industrialization and urbanization in developing countries have increased air pollution (Seinfeld 2004). Air pollution is a continuing challenge to public health because epidemiological studies have linked fine particulate matter (aerodynamic diameter: 2.5 μm; PM2.5) pollution to increasing negative effects on the human body, especially the respiratory system (Balashazy et al. 2003; Beelen et al. 2015; Pope et al. 2002, 2009; Shah et al. 2013). It is known that air pollution is generated by a variety of resource consumption, which is a heterogeneous mixture including metals, salts, carbonaceous material, volatile organic compounds, polycyclic aromatic hydrocarbons, and endotoxins. Because of the heterogeneity in the composition of PM, research is needed to evaluate the complex mechanisms underlying PM2.5-induced adverse health effects.

Non-small cell lung cancer (NSCLC) is the most common lung cancers in human, and less is known regarding the specific mechanisms through which PM2.5 exposure promotes NSCLC growth and progression. It is crucial to study the mechanisms in NSCLC after exposure to particulate matter to better characterize gene-environment interactions and epigenetic influences on cancer exacerbation.

In this study, we evaluated the effects induced in vitro by PM2.5 exposure on adenocarcinomic human alveolar basal cell line A549 and human non-small lung carcinoma cell line H1299. Their cancer properties were investigated by viability and monolayer wound healing assays. Furthermore, we performed bioinformatic analysis to uncover the mechanism and measured the levels of mRNAs and proteins implicated by in vitro experimental evidences of the toxic potential of PM2.5 to exacerbate cancer. In particular, we compared the effects on A549 and H1299 cells, focusing not only on migration, which was induced by PM2.5 exposure, but also their proliferation. The present study describes the intriguing process in which PM2.5-induced events contribute to the molecular and cellular mechanisms, and is informative for the prevention and treatment of air pollution-induced systemic diseases.

## Methods

### Experiment samples

The stock PM2.5 solution (5 mg/ml in PBS) was stored at −80 °C in our laboratory. PM2.5 samples were prepared and the analysis of PM2.5 composition was performed using the methods described previously (Ma et al. 2015). A549 and H1299 cells were grown in RPMI-1640 medium (Hyclone, USA) and kept at 37 °C in 5% CO_2_. The media were supplemented with 10% fetal bovine serum (Hyclone, USA) and 1% penicillin–streptomycin (Hyclone, USA). In vitro exposure to PM2.5 for 72 h 50 μg/cm^2^ and equal amount of PBS for 72 h, and then performing followed assays with conditioned supernatants (PM2.5-exposed supernatants and control supernatants) and treated cells (PM2.5-exposed cells and control cells).

### Cell proliferation assays

A549 and H1299 cells were seeded at a density of 1.0 × 10^4^ per well in triplicate in 96-well plates, and cultured in a CO_2_ incubator for 12 h before the medium was removed and replaced with two kinds of concentrations. Alternatively, in PM2.5 exposure group, cultured condition is composed of PM2.5-exposed supernatants and RPMI1640 absolute medium at a ratio of 1:3; in control group, cultured condition is composed of cultured medium and RPMI1640 absolute medium at a ratio of 1:3. Cell viability was assessed with CellTiter 96 AQueous One Solution Reagent (Promega, USA) according to the manufacturer’s recommendations. Absorbance was taken on a infinite M200 Pro Reader (TECAN, Switzerland) at a wavelength of 490 nm, at 0, 24, and 48 h. All assays were measured in triplicate from experiments performed 3 times.

### Monolayer wound healing assay

The A549 and H1299 cells were treated with 50 μg/cm^2^ concentrations of PM2.5 for 72 h, and then PM2.5-exposed cells were plated at a density of 2 × 10^6^ per well in six-well culture dish. The PM2.5-unexposed cells were seeded as control groups. Cells were then allowed to scratch approximately 300 μm width after 12 h with the 10 μl pipet tip (Gene Era Biotech, USA), at the end of which, The media and displaced cells were then removed, and cultured with 2% low serum culture medium. This procedure makes it possible to image the entire width of the wound using a 5 × objective (Leica AM6000, Germany). Photos were taken of the scratches, including the reference points, at 0, 24, and 48 h to monitor closure of the scratch. The migrated distance was calculated for 24 and 48 h based on the reduction of the scratch width with respect to the 0 h time point. Images are analyzed by digitally drawing lines (using Adobe Photoshop) averaging the position of the migrating cells at the wound edges.

### RNA isolation and quality control

After the A549 cells were treated with PM2.5 for 72 h, Total RNA was extracted using TRIZOL Reagent (Life technologies, USA) following the manufacturer’s instructions and checked for a RIN number to inspect RNA integrity by an Agilent Bioanalyzer 2100 (Agilent technologies, USA). RNA integrity was assessed by standard denaturing agarose gel electrophoresis. RNA concentration and RNA integrity were determined by capillary electrophoresis on an Agilent 2100 Bioanalyzer (Agilent Technologies, USA); only the samples with RNA integrity number more than 7 were used. RNA samples were stored at −80 °C until further processing.

### Gene expression data

Transcriptome high-throughput sequencing were performed at the Shanghai Biochip Co,. Ltd, according to the protocols in the HiSeq 2500 Sequencing System. Analyze the FPKM (Fragments Per Kilobase of exon model per Million mapped reads) values that was quantitative normalization, originated from the different genes on two samples using cufflinks. Differentially expressed genes were identified through Fold Change ≥2 and False Discovery Rate ≤0.05. The Gene Ontology (GO) project aims to describe gene and gene product attributes (http://www.geneontology.org), which covers three domains: biological process, cellular component and molecular function. Pathway analysis is a functional analysis that maps genes to Kyoto Encyclopedia of Genes and Genomes (KEGG) pathways (http://www.genome.jp/kegg/). The enrichment level was calculated by transforming the enrichment p values after False Discovery Rate (FDR) correction to negative log 10 values, and the lower the *p* value is the more significant the correlation (a p value cut-off is 0.05).

### QPCR

Quantitative real-time PCR (qRT-PCR) was performed in triplicate to detected the fold changes of candidate genes, using an ABI Prism 7500 (Applied Biosystems, USA) and SYBR Select Master Mix (Applied Biosystems, USA) according to the manufacturer’s instructions with the modification of total reaction volume being 20 μl. Primer pairs used for real-time PCR were shown in Table 1. GAPDH was used as an internal control and the expression of the target RNA was normalized to GAPDH. Thresholds for statistical significance are noted in Results. Relative expression levels were calculated using the 2^−ΔΔCt^ method.

**Table 1:** List of primers for RT-PCR

Gene	Forward	Reverse
TNFRSF1A	GGTCTCAACGCCATCCTG	GCTCCATTTATCAGAACATCTCC
TP53	ATGGCACTGAGGAAGATGCT	CAGATAATGCGGGAAAGAGG
EREG	ATGCCCGATGAGATCAACA	CGACAGGTTTCCCACATGAC
IL1A	GTGGGCTGTGCCAAGTGT	GGTCACGGTCAGGGTTGTA
IL1B	GAACTCCTGCTTCTCCTTGC	ACTTGGCACAGCCCACAG
MMP1	GCCTCTGATTGGTGAATGGT	TCTTGTCCCTCTGGTCCTGT
MT1X	TGGCAGAAAGGGAACAGAAA	CTGGCTGATGGACAGGAGAT
GAPDH	GAAGGTGAAGGTCGGAGTC	GAAGATGGTGATGGGATTTC

### Enzyme-linked immunosorbent assay

The A549 and H1299 cells were treated with 50 μg/cm^2^ concentrations of PM2.5 for 72 h, and then collected exposed culture supernatants. The PM2.5-unexposed culture supernatants were collected as control group. The production of IL1β and MMP1 in PM2.5 exposed group and control group was measured by ELISA. Respectively, 2 ml cultured supernatants from control group and exposure group were collected, and the supernatants were centrifuged for 10 min in speed of 3000 r/min, and then the supernatant was collected and preserved in −20 °C for preparation. The content levels of IL1β and MMP1 in supernatants were detected by using enzyme-linked immunosorbent assay (ELISA). The experimental procedures were carried out in strict according to the manufacturer’s instructions, and the kits were purchased from Thermo Fisher Scientific, USA.

### Protein network construction

In this study we used the protein–protein interactions from the STRING database, The integrative network of PM2.5-mediated non small cell lung cancer protein interactions was drawn, which integrates and weighs information from numerous sources, including activation, inhibition, binding, catalysis, reaction, and expression (Szklarczyk et al. 2011). The scores higher than 0.7 will be considered as high confidence, thus, we used the interactions with combined scores higher than 0.7 for further analysis.

### Statistical analysis

Experimental data were analyzed by using the software GraphPad Prism 6 (GraphPad Software, CA, USA). Data reported as mean ± SD of three independent experiments. ANOVA was used to evaluate the differences between groups. Statistical comparisons were made using an unpaired two-tailed Student’s *t* test for two groups. Differences were considered significant if p < 0.05.

## Results

### Effects of PM2.5 exposure on the proliferation of cancer cells

**Fig. 1:**
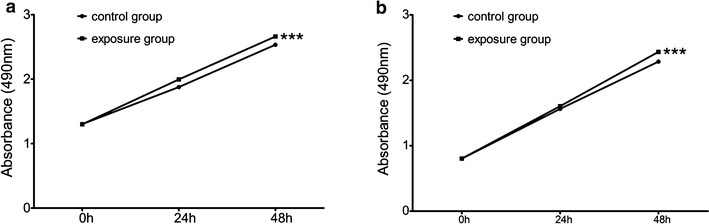
Effects of PM2.5-exposed culture supernatants on cancer cell proliferation. Data represent the mean ± SD of three independent experiments. ***p < 0.001 compared with control. **a** Proliferation of A549 cells in response to PM2.5-exposed culture supernatants treatment and control. **b** Proliferation of H1299 cells in response to PM2.5-exposed culture supernatants treatment and control

To investigate differences in the proliferation of NSCLC lines A549 and H1299 induced by PM2.5-exposed and unexposed culture supernatants, we performed cell proliferation assays. Compared with the unexposed culture supernatant group, the PM2.5-exposed culture supernatant group showed higher viability and proliferation (Fig. 1). Our results suggested that A549 and H1299 cells had larger populations after PM2.5 exposure. Therefore, under PM2.5-exposed circumstances, both A549 and H1299 cell lines had obviously strong ability on proliferation.

### Effect of PM2.5 exposure on cell migration

**Fig. 2:**
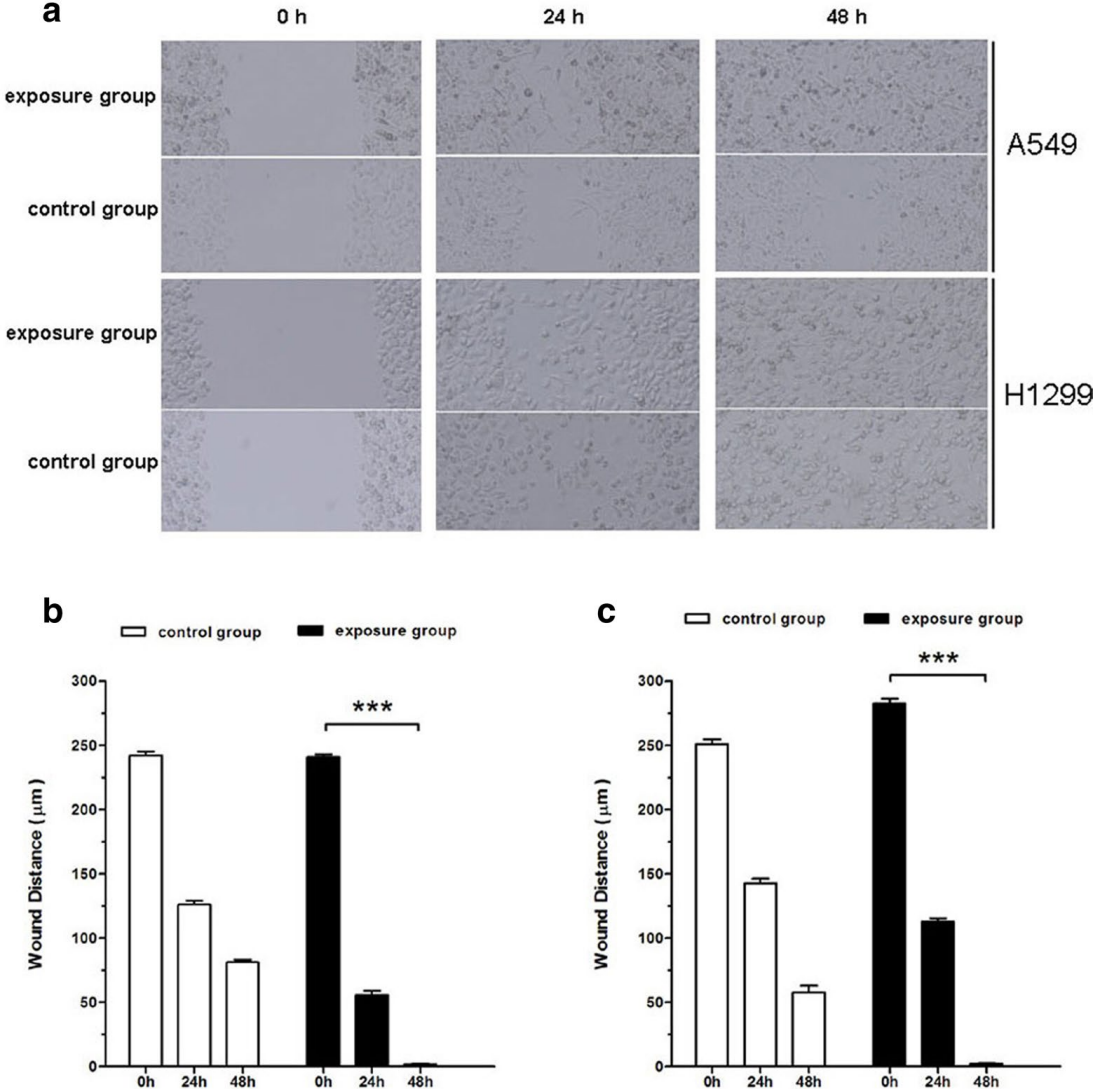
Effect of PM2.5 exposure on the migration of A549 and H1299 cells in monolayer scratch assays. **a** Phase contrast micrographs of the A549 and H1299 cells in exposure and control groups at various times after monolayer wounding. **b** Quantification of cell migration using the monolayer wound healing assay in A549 cells. **c** Quantification of cell migration using the monolayer wound healing assay in H1299 cells

We next investigated the effect of PM2.5 exposure on cancer cell migration using in vitro monolayer scratch assays. A549 and H1299 cells have high migratory and invasive abilities. The PM2.5-exposed cells had stable migration in contrast to the control group. Representative images of the scratches monitored over 48 h are shown in Fig. 2a. PM2.5 exposure increased the migration of cells compared with the untreated control cells. As shown in Fig. 2b, c, the monolayer was confluent in the PM2.5-exposed group at 48 h. Of note, compared with the PM2.5-exposed group, untreated cells migrated slower with significant differences at 24 and 48 h as indicated by repeated measures two-way analysis of variance.

### Transcriptome sequencing

**Fig. 3:**
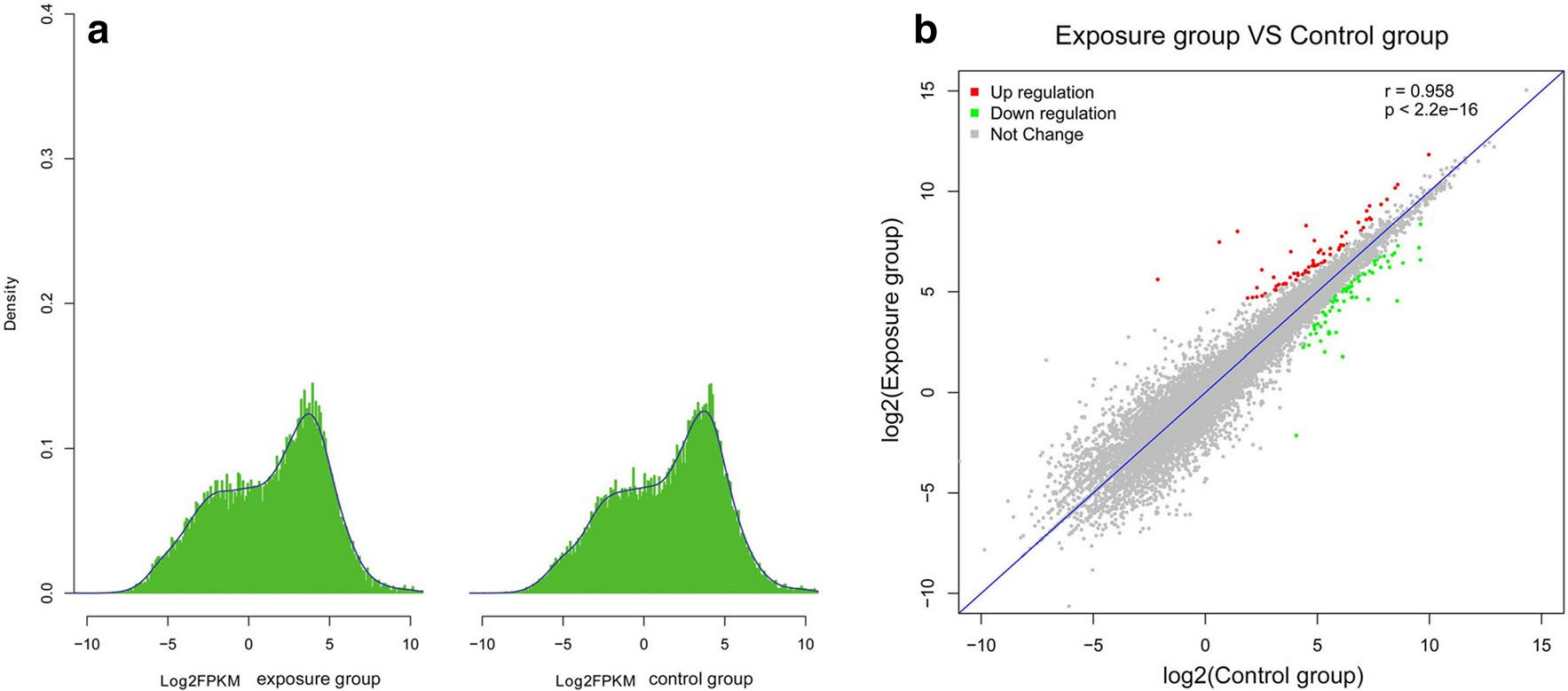
Gene expression profiling to characterize PM2.5 exposure. **a** Expression distribution plot. **b** Scatter plot of DEGs

Global gene expression profiling of A549 cells was performed using the Illumina microarray platform. By comparing mRNA expression profiles in the whole genome, we identified 143 differentially expressed genes (DEGs) in the exposure group relative to the control group (p < 0.05). After exposure to PM2.5 for 72 h, 22,389 mRNAs were identified in A549 cells. Among them, 143 genes with differential expression showed more than twofold changes, of which 66 genes were up-regulated and 77 were down-regulated (Fig. 3a, Expression distribution plot; Fig. 3b, Scatter plot). These results indicated that PM2.5 exposure produced a striking profile of DEGs compared with the control sample.

### Validation of gene regulations

**Fig. 4:**
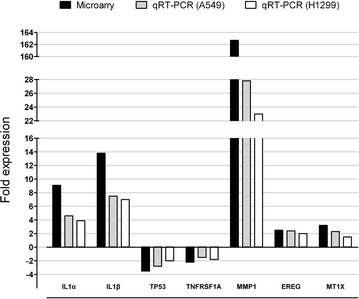
Correlation of transcriptional changes assayed by RNA-seq with those assayed by qRT-PCR. The GAPDH was used as an internal control to normalize target RNA

Next, to analyze the specific molecular mechanisms induced by PM2.5 exposure, some DEGs were subjected to further functional analyses. To validate mRNA expression levels of regulated transcripts, which responded to wound healing and MAPK signaling pathways, seven genes with significantly different expression were selected from the microarray data to be verified by quantitative RT-PCR (qRT-PCR) using cDNA from two experimental samples. These genes included five up-regulated genes [epiregulin (EREG), interleukin (IL) 1A, IL1B, matrix metalloprotease 1 (MMP1), and metallothionein 1X (MT1X)] and two down-regulated genes [tumor necrosis factor receptor superfamily 1A (TNFRSF1A) and tumor protein 53 (TP53)]. We found expression of these genes in the exposure group to significantly differ from their expression in the control group, similarly to the trends in the microarray data, as shown in Fig. 4. Our data demonstrated that the selected genes involved in proliferation and motility signaling showed significant differential expression. Among the selected genes, the level of MMP1 expression was the highest, indicated that motility of PM2.5-exposed cells was affected significantly.

### Secreted MMP1 and IL1β analysis by enzyme-linked immunosorbent assay

**Fig. 5:**
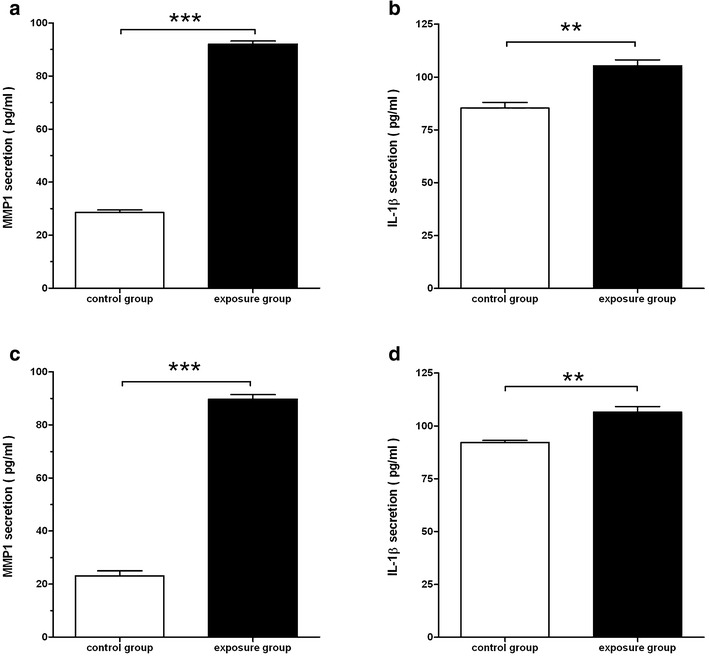
Determination of the contents in culture supernatants. **a**, **b** Content determination in the culture supernatants of A549 cells. **c**, **d** Content determination in the culture supernatants of H1299 cells

To determine whether the secreted levels of IL1β and MMP1 were higher in the exposure group than the control group, we performed enzyme-linked immunosorbent assays. The results are shown in Fig. 5. The secreted content of inflammatory mediators IL1β and MMP1 in the PM2.5-exposed group was obviously higher than that in the control group (p < 0.05). Combined with the results of functional analysis, these data showed that PM2.5 exposure had a significant influence on the expression of multiple inflammatory mediators. The results were similar to the qRT-PCR data that showed high gene expression of IL1β and MMP1 that are secreted in a paracrine fashion into the extracellular space.

### Network analysis of genes affected by PM2.5 exposure

**Fig. 6:**
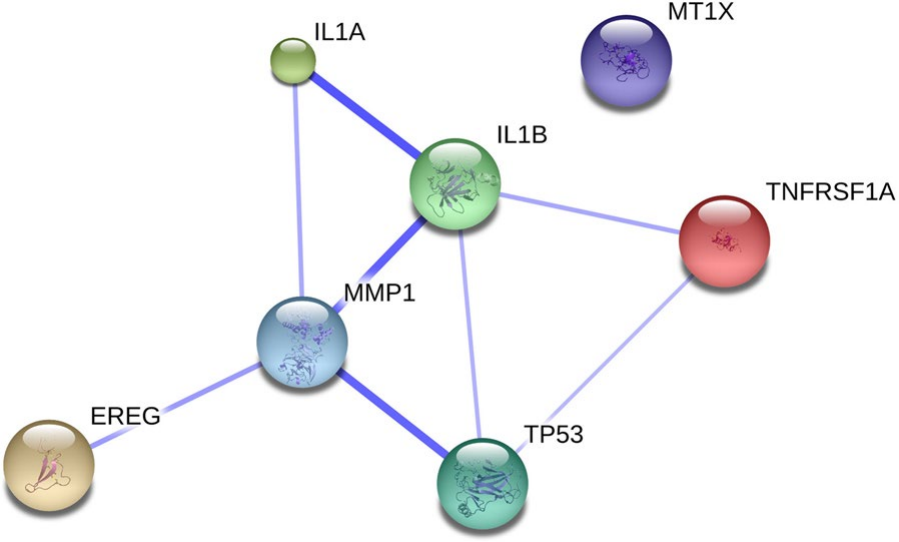
Interactive gene network view of PM2.5 exposure. The schematic represents a network to visualize PM2.5 exposure-related proteins

Using the String database, analysis of the selected genes revealed a significant association. The results of RNA-seq of PM2.5-exposed cells indicated enhancement of a network of seven interacting proteins (Fig. 6) that are strongly associated with connectivity and integration: IL1A, IL1B, MMP1, EREG, TNFRSF1A, and TP53. In the constructed network, IL1B, MMP1, TP53, and EREG played crucial roles, possibly because of the cell lines represent invasive and proliferative cancers, suggesting that the expression of these genes may be maintained in NSCLC after PM2.5 exposure.

## Discussion

Lung cancer is the leading cause of cancer-related death and thus a major health problem worldwide. Approximately 80% of lung tumors are NSCLC that includes squamous cell carcinoma, adenocarcinoma, and large cell carcinoma. The incidence of adenocarcinoma appears to be increasing worldwide (Khuder 2001; Risch and Plass 2008; Siegel et al. 2013). Particulate pollution is a serious environmental problem that is influencing air quality as well as regional and global climates, and epidemiological surveys show that air pollution may have negative effects on the human body, especially the respiratory system (Pope et al. 2002; Balashazy et al. 2003; Pope et al. 2009; Shah et al. 2013; Atkinson et al. 2014; Beelen et al. 2015). Additionally, numerous investigations on PM2.5 and human health were reported, mainly focusing on its cytotoxic effects (Laing et al. 2010; Ma et al. 2015; Deng et al. 2014) and effect of reactive oxygen species (Ni et al. 2015; Zuo et al. 2012; Jiang et al. 2014). The contributions of PM2.5, the main toxicological component of air pollution, to lung cancer are complex, and the specific mechanisms through which different genes regulate lung tumor growth and progression are not well defined. Therefore, focusing on careful characteristic evaluation of its toxic potential is still needed. In the present study, we demonstrate that PM2.5 induces more stronger motility and proliferation in A549 and H1299, and identified the key cellular and molecular factors, which may facilitate development of novel biomarkers of neoplastic progression and/or new therapeutic strategies for patients.

Our preliminary experiments indicated that PM2.5 efficiently induced proliferation in H292 tumor cells in vitro. In this context, we significantly expanded our previous findings and showed that induction of both proliferation and motility of tumor cells by PM2.5 exposure is controlled by different mechanisms that require regulators including mainly cytokines and MMPs. To examine whether cellular processes in NSCLC are either maintained or newly acquired after PM2.5 stimulation, A549 and H1299 cell lines were cultured in supernatants from cells exposed to PM2.5. As a result, we found that proliferation and the migration of exposed cells was stronger in viability assay and wound healing assay. These data imply that PM2.5 exposure induces cross-talk among pathways that promote survival, proliferation, invasion, and migration of tumor cells. Of note, as expected, key cellular processes associated with the response to wound healing and the mitogen-activated protein kinase (MAPK) signaling pathway played a crucial role.

Our study focused primarily on identifying important PM2.5-induced genes that contribute to pro-tumorigenic cell proliferation. To determine the relevance of these findings in human tumors, A549 and H1299 cell lines were analyzed for EREG, IL1A, IL1B, MMP1, MT1X, TP53 and TNFRSF1A expression. Under our experimental conditions, we found that PM2.5-induced genes play important roles in the regulation/promotion of cell proliferation. Initial studies of EREG, which is a member of the epidermal growth factor family, revealed that it is highly up-regulated in PM2.5-exposed cells in comparison with unexposed NSCLC cells. EREG promotes tumor cell survival, and previous studies have demonstrated that EREG is linked to pulmonary metastasis (Gupta et al. 2007). In this study, IL1β was the most up-regulated cytokine, which has a strong contribution to cell proliferation and an important function in cell–cell communication and can affect cellular functions (Chiang and Massagué 2008). Under the IL1β-stimulated condition, the NSCLC cell lines acquired obviously a proinflammatory signature that was dominant in the PM2.5-exposed group without losing invasive properties. In addition, EREG and IL1β act through induction of MMP1 to confer survival advantages. Our results led to identification of a novel pathway involving EREG and MMP-1, which contributes to the growth and progression of lung cancer. The IL1β-mediated pathway was associated with activation of MAPK signaling. Activation of the MAPK cascade can inactivate the p53 tumor suppressor, which can lead to sustained proliferation (Drosten et al. 2014). Moreover, our data demonstrated expression of TNFRSF1A, which encodes a subunit of the TNF-α receptor, and finally with the timing and duration of TNF-α signaling, down-regulation is capable of repressing apoptosis (Varfolomeev and Avi 2004). Taken together, these results facilitate molecular analysis of cell proliferation, which may contribute significantly to the level of proliferation in this study.

We also found that both the molecular signatures and network model provide a basis for revealing mechanisms of metastasis, and can be used to identify potential therapeutic targets for NSCLC after PM2.5 exposure. MMPs are involved in breakdown of the extracellular matrix, tissue healing, and remodeling (Martins et al. 2013; Nagase and Woessner 1999). In this study, MMP1 was the most up-regulated gene, and examination of EREG-induced signaling pathways has demonstrated that EREG promotes cell survival through regulating the expression of MMP1 (Boström et al. 2011; Farooqui et al. 2015). Our results also suggest that MMP1 has a strong ability to promote cancer invasion and metastasis. MMP1 decreases the oxygen consumption ratio, up-regulates the expression of hypoxia-inducible factor, and decreases the production of reactive oxygen species. In addition, MMP1 has been linked to the promotion of cell survival. The levels of MMP1 expression are associated with poor prognosis, suggesting that MMP1 may contribute to multiple processes during tumor growth and progression (Herrera et al. 2013). MMPs mediate expansion of cells, which often leads to angiogenesis (Ma et al. 2009). Similarly, IL1β can induce the formation of new vessels (Lee et al. 2011; Voronov et al. 2003). According these results, we confirmed that both IL1β and MMP1 were involved in the progression of cell invasion and metastasis.

This study provides a basis to identify key regulators responsible for the pathological features of NSCLC, demonstrating how a certain type of cell acquires a stronger functional proliferative/invasive potential under PM2.5 exposure. The pathological mechanisms have not been elucidated, and malignant transformation requires the acquisition of multiple phenotypes, such as proliferation, survival and migration, which contribute to tumor growth and progression (Hanahan and Weinberg 2011). However, our results demonstrate the dynamic and complex interplay of IL1A, IL1B, MMP1, TP53, and EREG in crucial roles through various mechanisms and cross-talk with other signaling pathways. In addition, we found elevated levels of MMPs and IL1 in supernatants, which modulate the microenvironment to stimulate tumor growth and invasion. The cardinal role of EREG signaling is promotion of cell survival, which contributes to increased tumor volume (Irmer et al. 2007; De Luca et al. 2011). Expression of EREG was significantly enhanced, leading to a possible mechanism in which high levels of EREG in the developing tumor microenvironment may enhance tumorigenic properties of the surrounding normal epithelium in a paracrine manner (Pedram et al. 1997). MMP-1 is associated with both cell survival and pre-invasive lesions, which was induced following EREG stimulation by PM2.5 exposure.

PM2.5 released from various sources may cause adverse effects on human health and on the environm ent. In each area, theeffective components is different in PM2.5, and the pathogenic mechanism of mixture also do not elide the distinction. Due to the heterogeneity in PM2.5 composition, it is different from the type of disease after exposure. It is necessary to carry out the related study, and the experiments of tumor bearing animal are performing in our laboratory.

## Conclusions

In this study, we observed that PM2.5 exposure induced proliferation and motility in A549 and H1299 cell lines. Our identified genes and networks are somewhat different from the well-known canonical pathways described so far, because the changes were generated by PM2.5 exposure. Our results revealed a novel changes that promote exacerbation of tumors after PM2.5 exposure. While further studies are required to fully implicate this pathway in promoting the development of lesions, they provide a foundation to better understand the complex interactions involved in promoting tumor progression.

## Abbreviations

PM2.5: particulate matter with an aerodynamic diameter less than 2.5 μm
NSCLC: non-small cell lung cancer
EREG: epiregulin
IL1A: interleukin 1, alpha
IL1B: interleukin 1, beta
MMP1: matrix metallopeptidase 1
MT1X: metallothionein 1X
TNFRSF1A: tumor necrosis factor receptor superfamily, member 1A
TP53: tumor protein p53
GAPDH: glyceraldehyde-3-phosphate dehydrogenase
MAPK signaling: mitogen-activated protein kinase signaling
FPKM: fragments per kilobase of exon model per million mapped reads
KEGG: Kyoto Encyclopedia of Genes and Genomes
GO: gene ontology
FDR: false discovery rate
DEGs: differentially expressed genes
